# Clinical markers predict the efficacy of several immune checkpoint inhibitors in patients with non-small cell lung cancer in China

**DOI:** 10.3389/fimmu.2023.1276107

**Published:** 2023-12-06

**Authors:** Yuxin Wang, Jiahui Lu, Chenxi Wu, Fei Fei, Zhuze Chu, Peihua Lu

**Affiliations:** ^1^ The Affiliated Wuxi People’s Hospital of Nanjing Medical University, Wuxi People’s Hospital, Wuxi Medical Center, Nanjing Medical University, Wuxi, China; ^2^ Department of Breast Surgery, The First Affiliated Hospital of Nanjing Medical University, Nanjing, China; ^3^ Department of Oncology, Wuxi People’s Hospital Affiliated to Nanjing Medical University, Wuxi, China

**Keywords:** non-small cell lung cancer, immune checkpoint inhibitors, neutrophil-to-lymphocyte ratio, CXCL13, CD8, biomarker, prognostic value

## Abstract

**Objectives:**

Immune checkpoint inhibitors (ICIs) are one of the most significant oncological treatment modalities as a result of the rapid advancement of immunotherapy. Programmed Cell Death-Ligand 1 (PD-L1) and tumor mutational burden (TMB) have emerged as key markers for predicting the efficacy and prognosis of ICIs in non-small cell lung cancer (NSCLC), and the predictive role of tumor-infiltrating lymphocytes (TILs) has also received significant attention. However, the prognosis of some individuals cannot be determined by these indicators; for instance, some patients with low PD-L1 expression also benefit from longer survival. Therefore, the purpose of this research was to investigate the connection between new haematological and pathological markers and clinical outcomes in NSCLC patients receiving ICIs.

**Methods:**

Seventy-six patients with stage III-IV NSCLC treated with ICIs were included in this study. We used the Mann-Whitney test, COX regression and Kaplan-Meier analysis to retrospectively analyze peripheral blood indicators and survival prognostic data of 76 patients in order to investigate the relationship between baseline neutrophil-to-lymphocyte ratio (NLR) and the efficacy of ICIs. To investigate the correlation between CXCL13, CXCR5, CD8 and the efficacy of ICIs, we assessed the expression levels of aforementioned indicators in biopsied tissues of 10 non-small cell lung tumors by immunohistochemistry (IHC) and immunofluorescence (IF) and performed statistical analysis.

**Results:**

Disease control rate (DCR) was higher in patients with baseline NLR <3.4 (p=0.016) and neutrophil percentage <71% (P=0.015). Baseline NLR (HR=2.364, P=0.003) and neutrophil percentage (HR=2.824, P=0.013) had the greatest influence on patients’ survival prognosis, with baseline NLR exhibiting a stronger predictive value (AUC=0.717), according to univariate and multifactorial COX regression analyses of progression-free survival (PFS) and overall survival (OS). In NSCLC tissues, higher expression of CXCL13 was associated with better clinical outcomes (P=0.032) and higher expression of CD8 was associated with prolonged survival (P=0.022).

**Conclusion:**

Low baseline NLR in peripheral blood and high expression of CD8 in tissues are associated with longer PFS and may have a potential predictive value for patients with stage III-IV NSCLC using ICIs.

## Introduction

Lung cancer is currently the malignant tumor with the highest mortality rate worldwide, and non-small cell lung cancer (NSCLC) is the most common tissue type among lung cancers (1). Many patients with NSCLC are already in advanced stages when they are discovered because of the insidious nature of tumors and the fact that early screening is not widely practiced (2). The current treatment for NSCLC mainly includes targeted therapies against oncogenic drivers and immunotherapy such as Immune checkpoint inhibitor (ICI) therapy and Chimeric Antigen Receptor (CAR) T-cell Therapy (CAR-T) (3). ICI therapy has been extensively studied as one of the major classes of immunotherapy and the predictive markers for their efficacy and prognosis are in full swing. The expression levels of Programmed Cell Death-Ligand 1 (PD-L1), tumor mutational burden (TMB), tumor-infiltrating lymphocytes (TILs) and gene expression profiles (GEP) are all recognized for their predictive role in the treatment of NSCLC with ICI, but all have its limitations (4, 5). Many patients with PD-L1 <1% have a substantial clinical benefit to ICI therapy (6), and TILs in the immune microenvironment require simultaneous observation by at least two pathologists and exists heterogeneity of observers (7). Therefore, it is important to explore more new predictive biomarkers.

Neutrophil-to-lymphocyte ratio (NLR) refers to the ratio between absolute peripheral blood neutrophil count and absolute lymphocyte count, which is a biomarker representing the balance between inflammation and anti-tumor immune response in the body (8). It can be easily obtained from routine blood tests and is highly clinically accessible. Baseline NLR refers to the NLR status within 2 weeks prior to the first ICI treatment and reflects the basal status *in vivo* prior to immunotherapy. The prognostic value of NLR for Nivolumab and Pembrolizumab in the treatment of NSCLC, gastric cancer, colorectal cancer, and melanoma has been studied, and higher NLR levels often represent a worse survival prognosis (9–12). However, compared with the early marketed ICIs such as Pembrolizumab and Nivolumab, the Chinese-developed ICIs such as Camrelizumab, Tislelizumab and Sintilimab have only been marketed and included in health insurance in the last five years, benefiting the majority of Chinese people, yet there are fewer studies on the efficacy of NLR to predict the above ICIs in NSCLC, so it is necessary to figure out a set of prognostic markers suitable for Chinese people. Camrelizumab, Tislelizumab and Sintilimab are all PD-1 inhibitors, of which Sindilizumab is the first PD-1 monoclonal antibody to enter China’s medical insurance catalog and also has the most first-line indications, including NSCLC. Camrelizumab has received a significant price cut after health insurance negotiations, and its emergence has pushed the price advantage of PD-1 inhibitors to new heights. Tislelizumab is also a PD-1 inhibitor independently developed by China. Studies have shown that the dissociation rate of Tislelizumab is 30 times slower than that of Nivolumab and 50 times slower than that of Pembrolizumab, which makes the affinity of Tislelizumab 30-50 times higher than that of the other two antibodies (13). Therefore, we performed this retrospective study to explore the prognostic value of NLR on NSCLC treated with the above-mentioned ICIs.

The CXCL13-CXCR5 axis is a chemokine ligand that regulates its activity by interacting with seven transmembrane G protein-coupled receptors (GPCRs), resulting in a chemokine ligand/receptor pair axis that has both pro-tumor and anti-tumor effects (14). It has been shown that patients with NSCLC have higher levels of CXCL13 and CXCR5 than the normal people (15, 16). CXCL13 CD8^+^ T cells were confirmed as poor prognostic factors for immunotherapy due to the presence of immune checkpoints such as Programmed cell death protein 1 (PD-1), but Mark Sorin et al. demonstrated by single cell spatial landscape that CXCL13 enhances the sensitivity of tumors to ICIs and has a positive prognostic effect (17). In addition, it was shown that five-year disease-free survival (DFS) was significantly lower in CXCR5-positive group of NSCLC patients, but after it was found that CXCL13 was able to recruit circulating CXCR5+ B cells and CXCR5+ CD4+ Follicular helper T (TFH) cell population to the intratumoral tertiary lymphoid structure (TLS), CXCR5+ CD8+ T cells showed greater proliferative capacity, more granzyme B production, tumor necrosis factor-α (TNF-α) and interferon-γ (IFN-γ) expression in different types of tumor tissues, thus specifically lysing tumor cells (18). Finally, CD8^+^T cell is a major component of TILs, which is also a good predictor of the prognosis of ICIs (19). Therefore, we evaluated the expression levels of CXCL13, CXCR5 and CD8 in tissues by Immunohistochemistry (IHC) and Immunofluorescence (IF) to explore the prognostic value of the above pathological markers for NSCLC treated with ICIs.

## Methods

### Patients and specimens

This retrospective study included 76 patients with stage III-IV NSCLC treated with ICIs at Wuxi People’s Hospital affiliated to Nanjing Medical University from November 2019 to July 2022. Basic clinical information and baseline hematological data were collected, and all patients were followed up until February 2023 with the aim of exploring the relationship between patients’ basic information and baseline hematological data and the relationship between the efficacy of ICIs. Ten of the 76 patients were randomly selected, and paraffin samples from their pre-treatment lung cancer tissue biopsies were requested for IHC and IF, with the aim of exploring the relationship between the expression levels of CXCL13, CXCR5, CD8 and the efficacy of ICIs in NSCLC tissues. This study was conducted with the consent of the Institutional Review Board of Wuxi People’s Hospital affiliated to Nanjing Medical University.

### Inclusion and exclusion criteria

Inclusion criteria: (i) all patients were over 18 years of age; (ii) diagnosis of NSCLC was confirmed by pathology; (iii) clinical stage was III/IV; (iv) baseline hematological data were obtained within 2 weeks before the first ICIs treatment. Exclusion criteria: (i) Patients were treated with ICIs for less than 3 courses; (ii) have undergone surgical treatment for NSCLC; (iii) severe side effects such as bone marrow suppression and impaired liver and kidney function have occurred during treatment.

### Clinical variables

Basic information of patients included gender, age, type of pathology, clinical stage, the expression level of PD-L1, kinds of ICIs, times of ICIs, treatment line, whether combined with chemotherapy, hypertension status and diabetes status. Baseline hematology data included white blood cell (WBC) count, lymphocyte count, monocyte count, neutrophil count, lymphocyte percentage, monocyte percentage, neutrophil percentage, baseline NLR (within 2 weeks prior to the first ICI treatment), later NLR (after the first course of treatment) and (WBC count-neutrophil count)/lymphocyte count (dNLR).

### Efficacy and survival assessment

Efficacy assessment can be categorized as complete remission, partial remission, stable disease, and disease progression, and the best outcome during the treatment of patients with ICIs was used as the final outcome assessment in this study. PFS is the time between the start of treatment and the detection of clinical/imaging progression or death from any cause. OS is the time between the start of treatment and death from any cause.

### Immunohistochemistry

Paraffin specimens from the pre-treatment biopsies of 10 patients were provided by the Department of Pathology, Wuxi People’s Hospital. We prepared four serial paraffin sections for each paraffin specimen using a sectioning machine, and the first one was stained with hematoxylin-eosin. We performed IHC for CXCL13 (dilution 1:500; Abcam). To analyze the immunohistochemical staining of CXCL13, the staining was independently evaluated by two investigators, based on a cell staining intensity score of 0-3, with 0 for no positive staining (negative), 1 for pale yellow (weakly positive), 2 for yellow (positive), and 3 for brown (strongly positive); the percentage of positive cells (0-100%) was calculated using ImageJ. The final score of CXCL13 immunohistochemistry was staining intensity × percentage of positive cells/3 random of high-power fields (HPFs) (400× magnification).

### Immunofluorescence

We performed Immunofluorescence double staining for CXCR5 (dilution 1:200; Abcam) and CD8 (dilution 1:200; Servicebio). We used orthomorphic fluorescence microscope (Nikon Eclipse C1) for observation under HPFs (200× magnification) and 3DHISTECH for shooting. The mean number of CXCR5+T and CD8+T cells/HPF was from 3 random fields and recorded by two investigators independently.

### Statistical analyses

IBM SPSS statistics version 24.0 was used for data analysis and GraphPad Prism 8.0.2 was used for graphic plotting. Clinical characteristics of patients were expressed as counts and percentages, and hematological data were expressed as medians and interval ranges. All independent clinical variables were included in the univariate COX regression, and those with P<0.2 were continued in the multivariate COX regression. The cutoff values of NLR and neutrophil percentage were confirmed using X-tile and were divided into high and low groups. Patients with disease progression (PD) were included in the no-benefit group and the remaining patients were included in the benefit group. The Mann-Whitney test was used to analyze the clinical and histological variables in the benefit and no-benefit groups. The chi-square test was used to analyze the relationship between the independent variables of clinical differences and efficacy. The receiver operating characteristic (ROC) curve was created to predict efficacy, and the Kaplan-Meier survival curves were used to analyze the differences in survival benefit. The chi-square test was used to perform basic information matching analysis. P < 0.05 was considered a statistically significant difference.

## Results

### Characteristics of patients

A total of 76 patients with advanced NSCLC treated with ICIs were enrolled in this study, with specific information in the **Table 1**. Among them, 68 were male, 8 were female, and 26 (34.2%) were younger than 65 years. Among the pathological types, adenocarcinoma accounted for the majority (44.8%), squamous carcinoma for 36.8% and the rest for 18.4%. 24 (31.6%) patients had stage III NSCLC and 52 (68.4%) had stage IV. In the case of ICI use, 32 patients (42.1%) used Camrelizumab, 22 (28.9%) used Tislelizumab, 17 (22.4%) used Sintilimab, 3 (3.9%) used Durvalumab, and 1 (1.3%) used Atezolizumab. The majority of patients who underwent 5-10 courses of ICI treatment were 39 (51.3%). The majority of patients (36, 47.4%) were treated with 1st-line ICI. Of the total number of patients, 71 (93.4%) had concurrent chemotherapy, 26 (34.2%) had hypertension, and 7 (9.2%) had diabetes. The median baseline NLR was 2.99 with a range of 0.48-8.65. The median Later NLR was 2.21 with a range of 0.95-6.98. The median dNLR was 1.59 with a range of 1.09-3.11. The median WBC count was 6.23×10^6^ with a range of (2.78-13.86)×10^6^. The median lymphocyte count was 1.37×10^6^ with a range of (0.44-2.75)×106. The median Monocyte count was 0.66×10^6^ with a range of (0.18-1.66)×10^6^. The median neutrophil count was 4.03×10^6^ with a range of (1.11-10.16)×10^6^. The median Lymphocyte percentage was 21.4% with a range of 9.6-60.1%. The median Monocyte percentage was 9.7% with a range of 1.8-20.1%. The median neutrophil percentage was 65.5% with a range of 29.1-83.4%. The median CD4/CD8 was 1.49 with a range of 0.49-3.81. The median score of CXCL13 was 26.25 with a range of 13.7-65.6. The median CXCR5 (cells/HPFs) was 6.17 with a range of 3.33-13. The median CD8 (cells/HPFs) was 13.84 with a range of 3-36.33.

**Table 1:** Basic information.

Basic information	Number
**Patients**	76
Sex	
Male	68 (89.5%)
Female	8 (10.5%)
Age	
<65	26 (34.2%)
≥65	50 (65.8%)
Histology	
Adenocarcinoma	34 (44.8%)
Squamous carcinoma	28 (36.8%)
others	14 (18.4%)
Clinical Stage	
III	24 (31.6%)
IV	52 (68.4%)
**PD-L1 status**	26
<1%	12 (46.1%)
≥1%	14 (53.9%)
Kinds of ICI	
Camrelizumab	32 (42.1%)
Tislelizumab	22 (28.9%)
Sintilimab	17 (22.4%)
Atezolizumab	1 (1.3%)
Durvalumab	3 (3.9%)
Times of ICI	
<5	22 (28.9%)
5-10	39 (51.3%)
≥10	15 (19.8%)
Treatment line	
1	36 (47.4%)
2	32 (42.1%)
≥3	8 (10.5%)
Combined chemotherapy	
No	5 (6.6%)
Yes	71 (93.4%)
Blood pressure status	
No	50 (65.8%)
Yes	26 (34.2%)
Diabetes status	
No	69 (90.8%)
Yes	7 (9.2%)
**Baseline NLR**×10^6^	2.99 (0.48-8.65)
**Later NLR**×10^6^	2.21 (0.95-6.98)
**dNLR**	1.59 (1.09-3.11)
**WBC count**×10^6^	6.23 (2.78-13.86)
**Lymphocyte count**×10^6^	1.37 (0.44-2.75)
**Monocyte count**×10^6^	0.66 (0.18-1.66)
**Neutrophil count**×10^6^	4.03 (1.11-10.16)
**Lymphocyte percentage×**100%	21.4 (9.6-60.1)
**Monocyte percentage**×100%	9.7 (1.8-20.1)
**Neutrophil percentage**×100%	65.5 (29.1-83.4)
**CD4/CD8**	1.49 (0.49-3.81)
**CXCL13** (score)	26.25 (13.7-65.6)
**CXCR5** (cells/HPFs)	6.17 (3.33-13)
**CD8** (cells/HPFs)	13.84 (3-36.33)

### Prognostic impact of clinical variables

Basic patient information and hematological data were included in a univariate COX regression (**Table 2**). For PFS, clinical stage (HR=1.642, P=0.123), times of ICIs (HR=0.425, P=0.041), treatment line (HR=0.428, P=0.117), baseline NLR (HR=1.225, P=0.029), later NLR (HR=1.253, P=0.022), neutrophil count (HR=1.110, P=0.149), lymphocyte percentage (HR=0.976, P=0.195) contributed to the prognosis (threshold: P<0.2). For OS, clinical stage (HR=2.511, P=0.062), times of ICIs (HR=0.142, P=0.003), baseline NLR (HR=1.179, P=0.146), later NLR (HR=1.282, P=0.045), neutrophil count (HR=1.143, P=0.142), lymphocyte percentage (HR=0.968, P=0.195), neutrophil percentage (HR=1.039, P=0.079) contributed to the prognosis (threshold: P<0.2). The X-tile software was used to determine the cutoff values of 3.4 for baseline NLR, 2.2 for later NLR, 3.5×10^6^ for neutrophil count, 18 for Lymphocyte percentage and 71 for neutrophil percentage. Multifactorial COX regression analysis showed that times of ICIs (HR=0.336, P=0.011) and baseline NLR (HR=2.364, P=0.003) were the two largest independent influencing factors for PFS (**Table 3**); clinical stage (HR=3.822, P=0.010), times of ICIs (HR=0.077. P=0.000), neutrophil count (HR=4.225, P=0.009) and neutrophil percentage (HR=2.824, P=0.013) were the four largest independent influencing factors for OS (**Table 4**).

**Table 2:** Univariate Cox regression of PFS and OS.

Variable	Category	PFS			OS		
		HR	95%CI	P-value	HR	95%CI	P-value
**Sex**	Male	Reference	–	–	Reference	–	–
	Female	0.644	0.256-1.623	0.351	1.141	0.395-3.294	0.807
**Age**	<60	Reference	–	–	Reference	–	–
	≥65	0.932	0.530-1.640	0.808	1.634	0.697-3.831	0.259
**Histology**	Adenocarcinoma	Reference	–	0.907	Reference	–	0.935
	Squamous carcinoma	0.906	0.488-1.682	0.754	1.167	0.511-2.666	0.714
	others	1.065	0.517-2.192	0.865	1.066	0.405-2.811	0.897
**Clinical Stage**	III	Reference	–	–	Reference	–	–
	IV	1.642	0.874-3.084	0.123	2.511	0.954-6.611	0.062
**PD-L1 status**	<1%	Reference	–	–	Reference	–	–
	≥1%	0.726	0.319-1.652	0.445	0.500	0.155-1.611	0.245
**Kinds of ICI**	Camrelizumab	Reference	–	0.779	Reference	–	0.602
	Tislelizumab	0.806	0.408-1.592	0.535	1.127	0.423-3.003	0.812
	Sintilimab	1.032	0.504-2.113	0.931	1.427	0.573-3.551	0.445
	Atezolizumab	1.194	0.160-8.911	0.863	5.049	0.631-40.393	0.127
	Durvalumab	1.846	0.551-6.184	0.320	0.867	0.114-6.615	0.890
**Times of ICI**	<5	Reference	–	0.080	Reference	–	0.003
	5-10	0.566	0.304-1.054	0.073	0.359	0.164-0.782	0.010
	≥10	0.425	0.187-0.966	0.041	0.142	0.039-0.511	0.003
**Treatment line**	1	Reference	–	0.292	Reference	–	0.680
	2	0.863	0.486-1.532	0.615	1.388	0.635-3.034	0.411
	≥3	0.428	0.148-1.237	0.117	0.998	0.278-3.588	0.997
**Combined chemotherapy**	No	Reference	–	–	Reference	–	–
	Yes	1.088	0.263-4.497	0.907	0.948	0.224-4.009	0.943
**Blood pressure status**	No	Reference	–	–	Reference	–	–
	Yes	0.902	0.504-1.612	0.727	1.098	0.516-2.339	0.808
**Diabetes status**	No	Reference	–	–	Reference	–	–
	Yes	0.933	0.370-2.353	0.884	.651	0.154-2.748	0.559
**Baseline NLR**	/	1.225	1.021-1.469	0.029	1.179	0.944-1.473	0.146
**Later NLR**	/	1.253	1.034-1.518	0.022	1.282	1.006-1.635	0.045
**dNLR**	/	0.961	0.432-2.141	0.923	0.539	0.153-1.897	0.336
**WBC count**	×10^6^	1.060	0.952-1.181	0.290	1.068	0.930-1.227	0.348
**Lymphocyte count**	×10^6^	0.983	0.552-1.752	0.954	0.978	0.470-2.032	0.952
**Monocyte count**	×10^6^	1.427	0.540-3.771	0.473	1.310	0.386-4.441	0.665
**Neutrophil count**	×10^6^	1.110	0.963-1.280	0.149	1.143	0.956-1.366	0.142
**Lymphocyte percentage**	×100%	0.976	0.940-1.013	0.195	0.968	0.922-1.017	0.195
**Monocyte percentage**	×100%	0.984	0.912-1.062	0.681	0.970	0.872-1.080	0.581
**Neutrophil percentage**	×100%	1.012	0.984-1.040	0.404	1.039	0.9961.084	0.079
**CD4/CD8**	/	0.768	0.481-1.226	0.268	0.801	0.430-1.491	0.484

**Table 3:** Multivariate Cox regression of PFS.

		HR	95%CI	P-value
**Times of ICI**	<5	Reference	–	0.024
	5-10	0.462	0.243-0.878	0.018
	≥10	0.336	0.145-0.780	0.011
**Baseline NLR**	Low (<3.4)	Reference	–	–
	High (≥3.4)	2.364	1.335-4.184	0.003

**Table 4:** Multivariate Cox regression of OS.

		HR	95%CI	P-value
**Clinical Stage**	III	Reference	–	–
	IV	3.822	1.382-10.570	0.010
**Times of ICI**	<5	Reference	–	0.000
	5-10	0.143	0.055-0.371	0.000
	≥10	0.077	0.019-0.306	0.000
**Neutrophil count**	Low (<3.5×10^6^)	Reference	–	–
	High (≥3.5×10^6^)	4.225	1.431-12.479	0.009
**Neutrophil percentage**	Low (<71%)	Reference	–	–
	High (≥71%)	2.824	1.194-6.679	0.013

**Figure 1:**
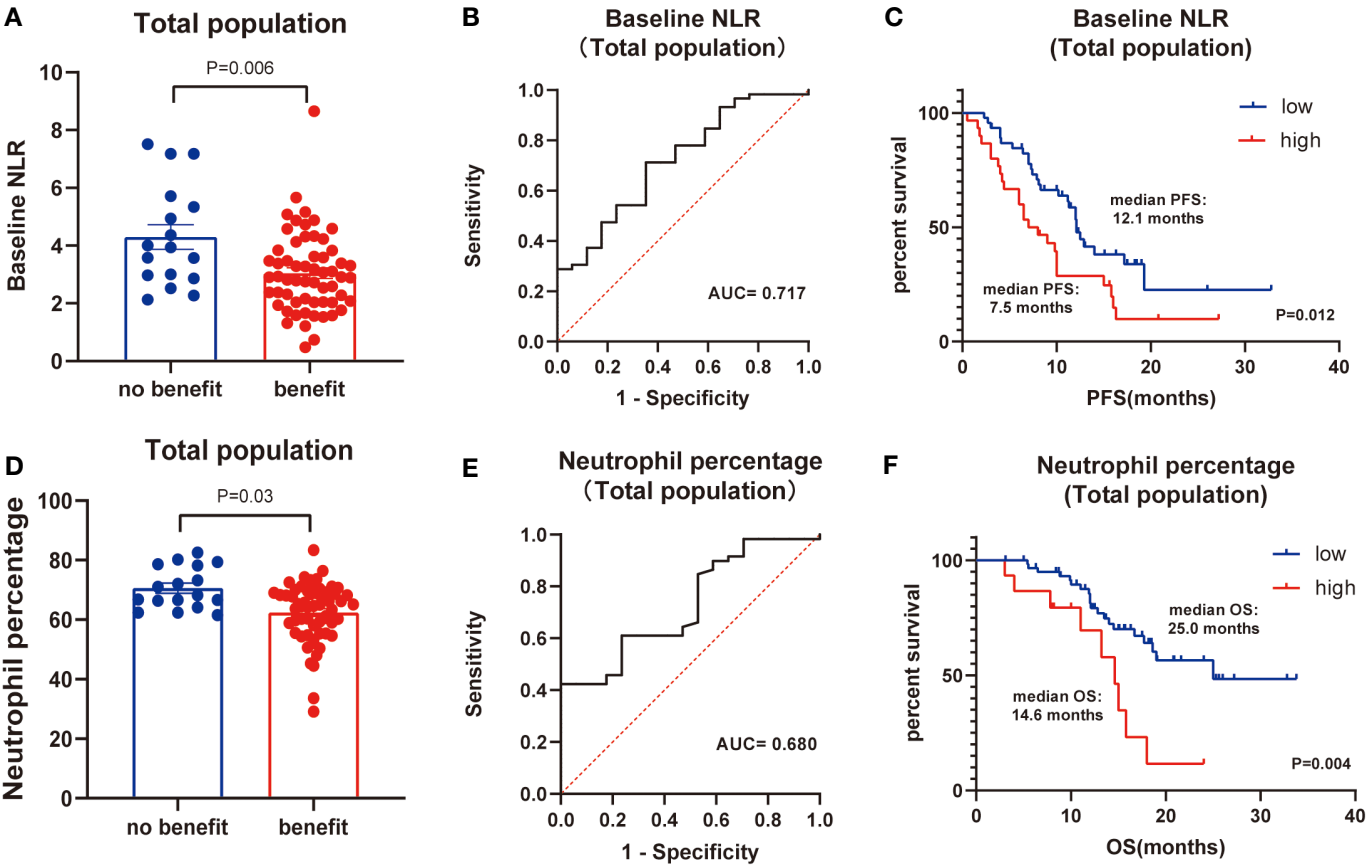
Total population analysis. **(A)** Differences of baseline NLR between benefit group and no-benefit group. **(B)** ROC curve of baseline NLR. **(C)** Kaplan-Meier survival curve of different levels of baseline NLR. **(D)** Differences of neutrophil percentage between benefit group and no-benefit group. **(E)** ROC curve of neutrophil percentage. **(F)** Kaplan-Meier survival curve of different levels of neutrophil percentage.

**Figure 2:**
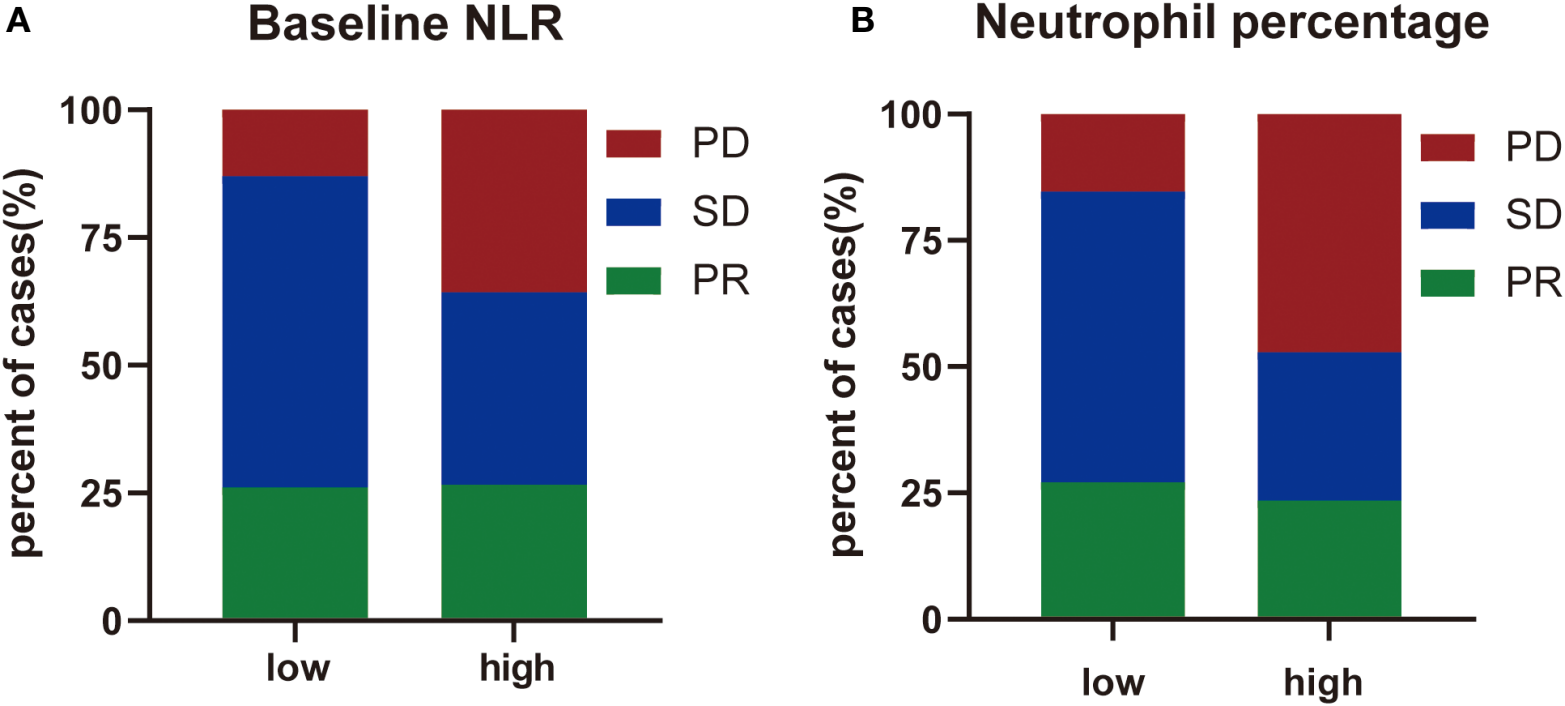
The distribution of treatment efficacy. **(A)** The distribution of treatment efficacy between high level group and low level group of baseline NLR. **(B)** The distribution of treatment efficacy between high level group and low level group of neutrophil percentage.

**Figure 3:**
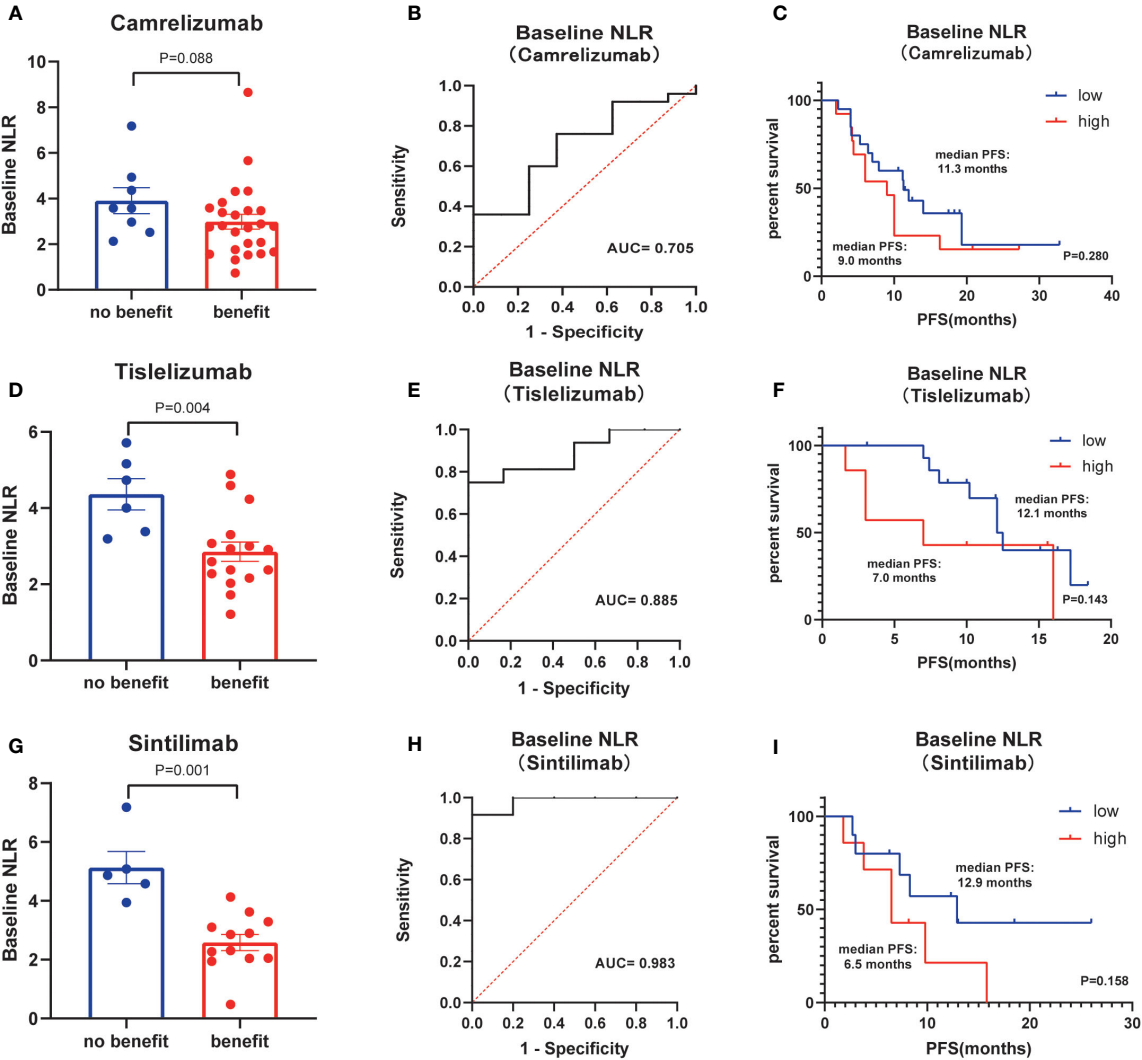
Subgroup analysis of kinds of ICIs. **(A)** Differences of baseline NLR between benefit group and no-benefit group among the ICI of Camrelizumab. **(B)** ROC curve of baseline NLR among the ICI of Camrelizumab. **(C)** Kaplan-Meier survival curve of different levels of baseline NLR among the ICI of Camrelizumab. **(D)** Differences of baseline NLR between benefit group and no-benefit group among the ICI of Tislelizumab. **(E)** ROC curve of baseline NLR among the ICI of Tislelizumab. **(F)** Kaplan-Meier survival curve of different levels of baseline NLR among the ICI of Tislelizumab. **(G)** Differences of baseline NLR between benefit group and no-benefit group among the ICI of Sintilimab. **(H)** ROC curve of baseline NLR among the ICI of Sintilimab. **(I)** Kaplan-Meier survival curve of different levels of baseline NLR among the ICI of Sintilimab.

Next, the differences in these factors above between the benefit and no-benefit groups were analyzed by the Mann-Whitney test, the baseline NLR was significantly lower in the benefit group than in the no-benefit group (P=0.006) (**Figure 1A**), and the neutrophil percentage was also lower in the benefit group than in the no-benefit group (P=0.03) (**Figure 1D**). However, the differences in the times of ICIs used and clinical stage between the benefit and no-benefit groups that were statistically significant in the multifactorial COX regression were not statistically significant. The receiver operating characteristic (ROC) curve for baseline NLR predicting PFS showed that an area under the curve (AUC) was 0.717 (**Figure 1B**), and the Kaplan-Meier survival curve showed that the low baseline NLR group had a better PFS benefit than the high baseline NLR group (p=0.012) (**Figure 1C**). The ROC curve for neutrophil percentage predicting OS showed that the AUC was 0.680 and the Kaplan-Meier survival curve showed that the low neutrophil percentage group had a better OS benefit than the high neutrophil percentage group (P=0.004) (**Figures 1E, F**). It is worth noting that disease control rates (DCR) were also better in patients with baseline NLR <3.4 and in patients with neutrophil percentage <71% (**Table 5**, **Figure 2**). Moreover, to investigate the effect of differential independent variables on efficacy in different ICI subgroups, a Mann-Whitney test was performed and found that baseline NLR levels were significantly lower in the benefit group than in the no-benefit group in both the Tislelizumab subgroup (P=0.004) and the Sintilimab subgroup (P=0.001) (**Figures 3D, G**). Unfortunately, other analyses in the ICI subgroup were not statistically significant (**Figures 3A–C, E, F**).

**Table 5:** Comparing treatment efficacy between two groups.

	Baseline NLR<3.4	Baseline NLR≥3.4	P-value	Neutrophil percentage<71%	Neutrophil percentage≥71%	P-value
**CR**	0 (0%)	0 (0%)	–	0 (0%)	0 (0%)	–
**PR**	12 (26.1%)	8 (26.6%)	–	16 (27.1%)	4 (23.5%)	–
**SD**	28 (60.9%)	11 (36.7%)	–	34 (57.6%)	5 (29.4%)	–
**PD**	6 (13.0%)	11 (36.7%)	–	9 (15.3%)	8 (47.1%)	–
**DCR**	40 (87.0%)	19 (63.3%)	0.016	50 (84.7%)	9 (52.9%)	0.015

**Figure 4:**
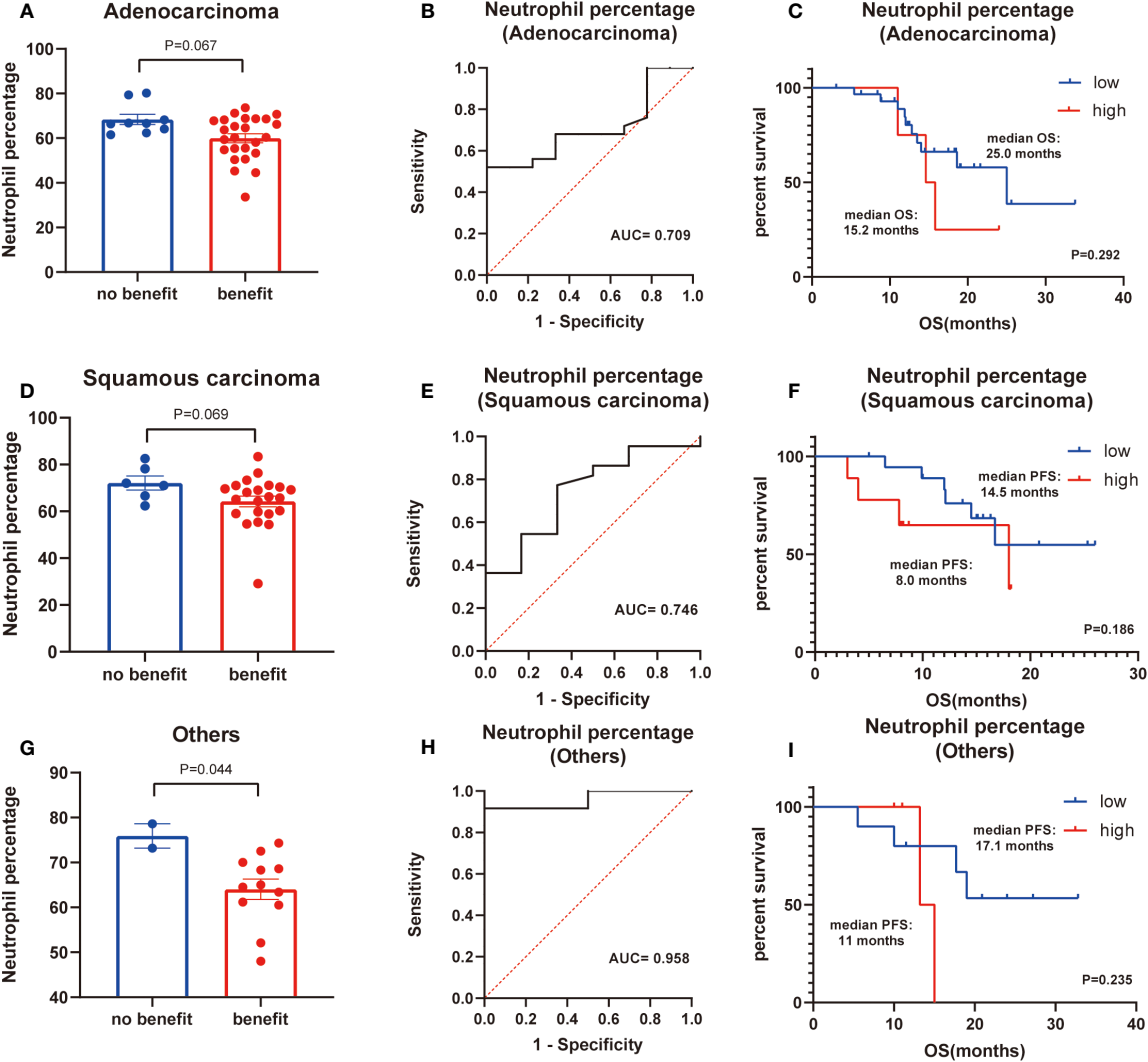
Subgroup analysis of histology of NSCLC. **(A)** Differences of neutrophil percentage between benefit group and no-benefit group among the adenocarcinoma. **(B)** ROC curve of neutrophil percentage among the adenocarcinoma. **(C)** Kaplan-Meier survival curve of different levels of neutrophil percentage among the adenocarcinoma. **(D)** Differences of neutrophil percentage between benefit group and no-benefit group among the squamous carcinoma. **(E)** ROC curve of neutrophil percentage among the squamous carcinoma. **(F)** Kaplan-Meier survival curve of different levels of neutrophil percentage among the squamous carcinoma. **(G)** Differences of neutrophil percentage between benefit group and no-benefit group among the other types of NSCLC. **(H)** ROC curve of neutrophil percentage among the other types of NSCLC. **(I)** Kaplan-Meier survival curve of different levels of neutrophil percentage among the other types of NSCLC.

Finally, basic information matching analysis by chi-square test revealed that baseline NLR matched all basic information, while neutrophil percentage was strongly correlated with the pathological type of NSCLC (P=0.001) (**Table 6**). The difference in neutrophil percentage between the benefit and no-benefit groups was not significant in both adenocarcinoma (P=0.067) and squamous carcinoma (P=0.069) (**Figures 4A, D**), the ROC curves showed AUC of 0.709 and 0.746 (**Figures 4B, E**), and Kaplan-Meier survival curves also showed that there was no significant prognostic difference between high level neutrophil percentage and low level neutrophil percentage in adenocarcinoma (P=0.292) and squamous carcinoma (P=0.186) (**Figures 4C, F**). In other types of NSCLC, the difference in neutrophil percentage between the benefit and no-benefit groups was statistically different (P=0.044) (**Figure 4G**), and the ROC curve showed an AUC of 0.958 (**Figure 4H**), but the Kaplan-Meier survival curve showed that high level neutrophil percentage and low level neutrophil percentage did not have a significant prognostic difference in other types of NSCLC (P=0.235) (**Figure 4I**).

**Table 6:** Basic information matching analysis.

	Baseline NLR		P-value	Neutrophil percentage		P-value
	Low	High		Low	High	
**Patients**	46	30		60	16	
**Sex**			0.794			1.000
Male	42(91.3%)	26(86.7%)		53(89.8%)	15(88.2%)	
Female	4(8.7%)	4(13.3%)		6(10.2%)	2(11.8%)	
**Age**			0.532			0.636
<65	17(37.0%)	9(30%)		21(35.6%)	5(29.4%)	
≥65	29(63.0%)	21(70%)		38(64.4%)	12(70.6%)	
**Histology**			0.091			0.001
Adenocarcinoma	25(54.3%)	9(30.0%)		30(50.8%)	4(14.8%)	
Squamous carcinoma	15(32.6%)	13(43.3%)		19(32.2%)	9(33.3%)	
others	6(13.1%)	8(26.7%)		10(17.0%)	14(51.9%)	
**Clinical Stage**			0.790			0.119
III	14(30.4%)	10(33.3%)		16(27.1%)	8(47.1%)	
IV	32(69.6%)	20(66.7%)		43(72.9%)	9(52.9%)	
**Kinds of ICI**			0.214			0.167
Camrelizumab	20(43.5)	13(43.4%)		29(49.2%)	4(23.5%)	
Tislelizumab	15(32.6%)	7(23.3%)		16(27.1%)	6(35.3%)	
Sintilimab	10(21.7%)	7(23.3%)		12(20.3%)	5(29.4%)	
Atezolizumab	1(2.2%)	0(0.0%)		1(1.7%)	0(0.0%)	
Curvalumab	0(0.0%)	3(10.0%)		1(1.7%)	2(11.8%)	
**Times of ICI**			0.981			0.600
<5	13(28.3%)	9(30%)		16(27.1%)	6(35.3%)	
5-10	24(52.2%)	15(50%)		30(50.8%)	9(52.9%)	
≥10	9(19.5%)	6(20%)		13(22.1%)	2(11.8%)	
**Treatment line**			0.204			0.276
1	22(47.8%)	14(46.7%)		27(45.8%)	9(52.9%)	
2	17(37.0%)	15(50%)		24(40.7%)	8(47.1%)	
≥3	7(15.2%)	1(3.3%)		8(13.5%)	0(0.0%)	
**Combined chemotherapy**			1.000			1.000
No	3(6.5%)	2(6.7%)		4(6.8%)	1(5.9%)	
Yes	43(93.5%)	28(93.3%)		55(93.2%)	16(94.1%)	
**Blood pressure status**			0.532			0.292
No	29(63.0%)	21(70.0%)		37(62.7%)	13(76.5%)	
Yes	17(37.0%)	9(30.0%)		22(37.3%)	4(23.5%)	
**Diabetes status**			1.000			0.950
No	42(91.3%)	27(90.0%)		53(89.8%)	16(94.1%)	
Yes	4(8.7%)	3(10.0%)		6(10.2%)	1(5.9%)	

### Prognostic impact of pathological markers

**Figure 5:**
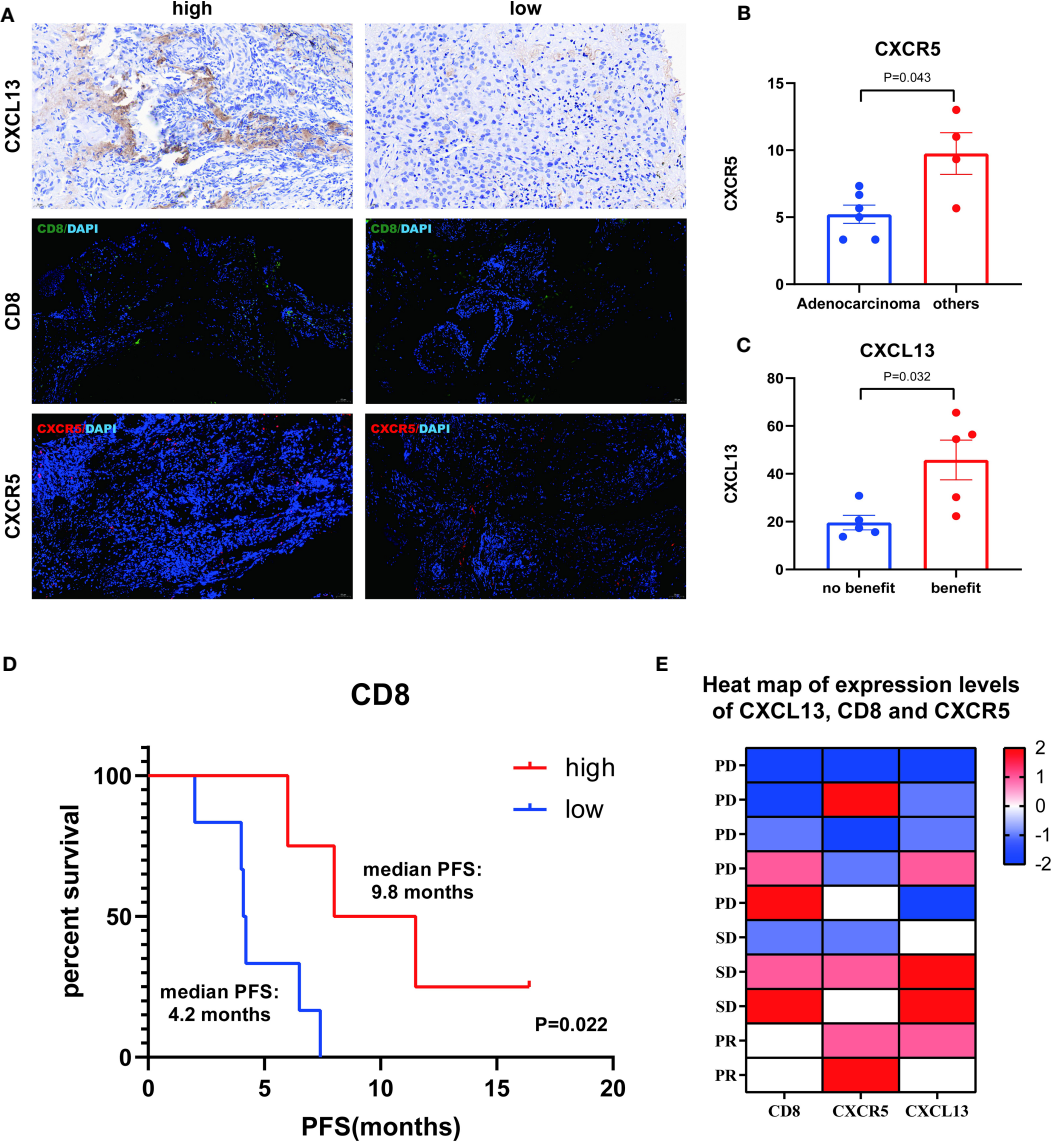
Analysis of CXCL13, CD8 and CXCR5. **(A)** Representative IHC and IF images of high and low expression of CXCL13, CD8 and CXCR5. **(B)** Differences of CXCR5+ cells between adenocarcinoma group and other cancer group. **(C)** Differences of CXCL13+ cells between benefit group and no-benefit group. **(D)** Kaplan-Meier survival curve of different levels of CD8+ cells. **(E)** Heat map of expression levels of CXCL13, CD8 and CXCR5.

To identify the presence of CXCR5, CD8, and CXCR5 in NSCLC, we performed immunohistochemistry and double immunofluorescence staining (**Figure 5**). CXCR5 expression was found to be lower in the adenocarcinoma group than in the other cancer groups by the Mann-Whitney test (P=0.043) (**Figure 5B**), CXCL13 was higher in the benefit group than in the no-benefit group (P=0.032) (**Figure 5C**), and a better PFS benefit was found in the high CD8 group than in the low CD8 group by the Kaplan-Meier survival curve (P=0.022) (**Figure 5D**). The heat map shows the levels of expression of different pathological markers in the 10 samples (**Figure 5E**). The expression levels of CD8, CXCR5 and CXCL13 were generally higher in the partial response (PR) and stable disease (SD) groups than in the PD group, and there was some consistency in the expression levels of the three pathological markers.

## Discussion

Lung cancer remains the number one cancer with the highest mortality rate worldwide, and NSCLC is the most dominant type of lung cancer, the importance and severity of which cannot be overstated (20). With the advent of immunotherapy, the treatment of NSCLC is becoming more and more diversified and targeted, and ICIs are developing more and more rapidly among them (21). Pembrolizumab and Nivolumab were approved for marketing as early as 2014, and studies on the efficacy and prognosis of these two ICIs have been very comprehensive (22), while Camrelizumab, Tislelizumab, and Sintilimab developed and launched by China were approved for marketing only in the last five years, and studies on their efficacy and prognosis still need to be conducted.

While many patients benefit from ICI therapy such as Camrelizumab, there are some patients who do not respond to ICI therapy or whose disease progresses quickly after treatment, so it is important to tap into clear predictive markers. The main biomarkers for predicting ICI efficacy are PD-L1, TMB, and microsatellite instability (MSI), of which only PD-L1 is approved for clinical prediction (23). However, approximately 15% of PD-L1-negative patients have clinical benefit and 40% of PD-L1-positive patients have no clinical benefit (24). Therefore, it is necessary to explore more potential biomarkers.

NLR is an important blood inflammatory marker that has been shown to be associated with the prognosis of a variety of tumors, asthma, and chronic obstructive pulmonary disease (COPD) (25, 26), but the prognosis of Camrelizumab, Tislelizumab, and Tislelizumab for NSCLC has been less well studied. In addition, the role of neutrophils on tumors has been extensively studied, and it has a dual role in cancer (27). On the one hand, N1-type neutrophils have high immune activity and promote the activation of CD8^+^ T cells to induce tumor cell killing; on the other hand, N2-type neutrophils can release matrix metalloproteinase 9 (MMP 9) to promote angiogenesis and spread of tumor cells (28). Therefore, studying neutrophil counts and percentages alone is also very important for the prognosis of NSCLC treated with ICIs. Our study found a statistically significant effect of baseline NLR and neutrophil percentage on the prognosis of NSCLC. First, we listed the basic information of 76 clinical patients, and then screened independent influences on prognosis by univariate and multifactorial COX regression. In addition, the Mann-Whitney test analyzed the differences between the benefit and no-benefit groups for indicators that were statistically different in the multifactor COX regression. Unfortunately, only baseline NLR and neutrophil percentage were statistically significant, so we focused on the prognostic significance of baseline NLR and neutrophil percentage for survival. Kaplan-Meier curves showed a median PFS of 12.1 months in the low baseline NLR level group and 7.5 months in the high baseline NLR level group, with a statistically significant difference between the two (P=0.012); median OS of 25.0 months in the low neutrophil percentage level group and 14.6 months in the high neutrophil percentage level group, with a statistically significant difference between the two (P=0.004). This also suggested that low baseline NLR level and low neutrophil percentage level are independent influences on the positive prognosis of NSCLC treated with ICIs. All these statistically significant results provided a good basis for follow-up studies.

The pro-cancer and anti-cancer effects of CXCL13-CXCR5 axis on NSCLC still remain to be studied (29), and some scholars have pointed out that it is a poor prognostic factor for immunotherapy because of the presence of PD-1 on the surface of CXCL13^+^ T cells and CXCR5^+^ T cells (30). However, from the analysis of the principle of ICI therapy, the PD-L1 inhibitor in ICIs binds to the immune checkpoint PD-L1, thus reducing the binding of PD-L1 to PD-1, which may offset the adverse effect of increased PD-1 expression (31), and CXCL13 can also enhance the sensitivity of tumors to ICIs (17), so the prognostic role of CXCL13 and CXCR5 deserves to be further investigated. In addition, CD8+ T cells are one of the most important immune cells of the organism and its expression level in tissues deserves to be explored (32). Kaplan-Meier curves showed a median PFS of 9.8 months in the high CD8 level group and 4.2 months in the low CD8 level group, with a statistically significant difference between the two (P=0.022). We also found significant differences in CXCL13 between the clinical benefit and no-benefit groups. These statistically significant results also provided a good basis for follow-up large-sample studies.

Many studies have confirmed that the combined predictive effect is more accurate than single prediction. Although only 10 pathological samples of enrolled patients were included in this study so far, the preliminary analysis of CXCL13, CXCR5, and CD8 has been statistically significant, and we will further expand the sample size in the future to further investigate the accuracy and validity of the combined prediction of peripheral blood markers NLR and pathological markers.

The study also had a few drawbacks. First of all, it was a retrospective study, and even if the principle of randomization was used, bias was unavoidable. Some patients did not reach the endpoint and still required additional follow-up, necessitating the need for more prospective studies in the future to fill these gaps. In addition, the total sample size included in this study was small due to the limited number of patients treated with more than 2 ICI sessions in a given time period at one hospital, and future multi-center studies and additional validation cohorts could be conducted. It is worth noting that the number of pathology samples included in this study was small due to sample accessibility and other issues, which may lack a certain degree of representativeness, and this deficiency can be addressed in subsequent prospective studies.

## Conclusion

Our findings suggest that in hematological data, baseline NLR and neutrophil ratio are strong predictors of outcome and prognosis in NSCLC patients treated with ICIs. This result is very indicative of the importance of studying the efficacy of NLR for predicting ICIs in NSCLC locally in China. In histological samples, CXCL13 expression was higher in the clinical benefit group than in the no-benefit group, and CD8 expression levels were strongly predictive of NSCLC patients treated with ICIs. Therefore, in the future, we have to further develop the joint prediction of NLR and CXCL13, CD8 and other indicators, establish a joint prediction model, and figure out a prognostic prediction model suitable for Chinese people. Several of these indicators have the potential to be effective biomarkers, and we need further prospective studies to demonstrate them.

## Data availability statement

The raw data supporting the conclusions of this article will be made available by the authors, without undue reservation.

## Ethics statement

The studies involving humans were approved by the Institutional Review Board of Wuxi People’s Hospital Affiliated to Nanjing Medical University. The studies were conducted in accordance with the local legislation and institutional requirements. The participants provided their written informed consent to participate in this study.

## Author contributions

YW: Writing – original draft. JL: Writing – original draft. CW: Writing – original draft. FF: Writing – original draft. ZC: Writing – original draft. PL: Writing – review & editing.
